# LG24 and sex chromosomes

**DOI:** 10.18632/oncotarget.5250

**Published:** 2015-08-24

**Authors:** Rong Huang, Zuoyan Zhu, Yaping Wang

**Affiliations:** State Key Laboratory of Freshwater Ecology and Biotechnology, Institute of Hydrobiology, Chinese Academy of Sciences, Wuhan, Hubei, China

**Keywords:** Chromosome Section, grass carp, whole-genome sequencing, chromosome fusion, sex determination

The grass carp (*Ctenopharyngodon idella*) is one of the most important freshwater fish species in China and the world; grass carp production accounts for about 16% of the world's total freshwater aquaculture [1]. In 2015, Wang et al. performed whole-genome sequencing and de novo assembly for a female (0.9 Gb) and male grass carp (1.07 Gb) by using the shotgun sequencing strategy [2]. Based on the transcriptome data for the grass carp and annotation information for the zebrafish (*Danio rerio*), 27,263 protein-coding genes were annotated in the grass carp genome. Although no sex chromosomes have been identified in the grass carp, genome comparative analyses of female grass carp, male grass carp, and other vertebrates provide important clues for studying the mechanism of underlying sex determination in the grass carp.

To analyze the evolutionary relationship between the grass carp and other species (*Homo sapiens*, *Mus musculus*, *Gallus gallus*, *Anolis carolinensis*, *Xenopus tropicalis*, *Petromyzon marinus*, *D. rerio*, *Gasterosteus aculeatus*, *Tetraodon nigroviridis*, *Takifugu rubripes*, *Oryzias latipes*, and *Gadus morhua*), we constructed a phylogenetic tree based on 202 single-copy genes with one-to-one correspondence in the different genomes. Phylogenetic tree analysis showed that the genetic relationship between the grass carp and zebrafish was the closest, and their differentiation time was about 49–54 million years ago. Comparative analysis of gene families among different species showed that the grass carp and zebrafish share 7,227 gene families and that most of the gene families had the same copy numbers. The grass carp and zebrafish experienced similar whole-genome duplication processes during evolution [2].

Using a genetic linkage map for the grass carp [3], 114 scaffolds were assembled into 24 linkage groups, which corresponded to 24 chromosomes of the grass carp and covered 64% of the genome [2]. Gene synteny analysis of the 24 linkage groups in the grass carp and 25 chromosomes in the zebrafish showed that most of the grass carp linkage groups corresponded to the zebrafish chromosomes, while LG22 and LG24 of the grass carp have undergone significant cross-chromosome arrangement. It is worth noting that the two ends of LG24 in the grass carp were compared to No. 10 and No. 22 chromosomes of the zebrafish, respectively. Fluorescence in situ hybridization confirmed that two grass carp markers corresponding to No. 10 and No. 22 chromosomes of the zebrafish were both located in LG24 in the grass carp [2]. This result suggested that the grass carp genome underwent chromosome fusion during evolution.

Comparative analysis of the female and male genomes of the grass carp was performed, and 2.38 Mb male-specific sequences, including 206 contigs, were identified. A total of 111 genes were predicted on the 206 contigs, 40 genes had homologs in the female genome, and 22 of the 40 genes were located in LG24. Physical length of LG24 was the greatest, and the genetic distance was the shortest; this showed that the exchange rate is significantly lower during meiosis. We know that the zebrafish has 25 pairs of chromosomes (*n* = 25), no sex chromosome differentiation, and an autosome-related sex-determination type [4]. The grass carp has 24 pairs of chromosomes (n = 24), experienced sex chromosome differentiation, and has the X/Y sex-determination type [5, 6]. Although we have not yet identified the sex chromosomes of the grass carp, the results suggest that chromosome fusion of LG24 may be related to sex chromosome differentiation in the grass carp.
